# Deletion of soluble epoxide hydrolase suppressed chronic kidney disease-related vascular calcification by restoring Sirtuin 3 expression

**DOI:** 10.1038/s41419-021-04283-6

**Published:** 2021-10-23

**Authors:** Wanbing He, Jieping Huang, Yang Liu, Changming Xie, Kun Zhang, Xinhong Zhu, Jie Chen, Hui Huang

**Affiliations:** 1 Department of Cardiology, Sun Yat-sen Memorial Hospital, Sun Yat-sen University, 107 Yanjiang Road, Guangzhou, 510120 China; 2 Department of Cardiology, The Eighth Affiliated Hospital of Sun Yat-sen University, 3025 Shennan Middle Road, Shenzhen, 518033 China; 3 Department of Cardiology, The Second Affiliated Hospital, University of South China, 30 Jiefang Road, Hengyang, 421001 China; 4 Research Center of Brain Health, Pazhou Lab, 70 Anyue Road, Guangzhou, 510330 China; 5 Department of Radiotherapy, Sun Yat-sen Memorial Hospital, Sun Yat-sen University, 107 Yanjiang Road, Guangzhou, 510120 China

**Keywords:** Chronic kidney disease, Calcification

## Abstract

Vascular calcification is common in chronic kidney disease (CKD) and contributes to cardiovascular disease (CVD) without any effective therapies available up to date. The expression of soluble epoxide hydrolase (sEH) is different in patients with and without vascular calcification. The present study investigates the role of sEH as a potential mediator of vascular calcification in CKD. Both Ephx2^−^^/−^ and wild-type (WT) mice fed with high adenine and phosphate (AP) diet were used to explore the vascular calcification in CKD. Compared with WT, deletion of sEH inhibited vascular calcification induced by AP. sEH deletion also abolished high phosphorus (Pi)-induced phenotypic transition of vascular smooth muscle cells (VSMCs) independent of its epoxyeicosatrienoic acids (EETs) hydrolysis. Further gene expression analysis identified the potential role of Sirtuin 3 (Sirt3) in the sEH-regulated VSMC calcification. Under high Pi treatment, sEH interacted with Sirt3, which might destabilize Sirt3 and accelerate the degradation of Sirt3. Deletion of sEH may preserve the expression of Sirt3, and thus maintain the mitochondrial adenosine triphosphate (ATP) synthesis and morphology, significantly suppressing VSMC calcification. Our data supported that sEH deletion inhibited vascular calcification and indicated a promising target of sEH inhibition in vascular calcification prevention.

## Introduction

Vascular calcification is a highly regulated process sharing a similar mechanism with bone formation [1]. It is commonly observed in aging and chronic diseases such as chronic kidney disease (CKD) [2]. Emerging evidence indicates that vascular calcification contributes to the high cardiovascular mortality and morbidity, especially in patients with CKD. Consistently a high phosphorus (Pi) level resulting from dysregulated mineral balance in CKD drives the occurrence of vascular calcification [1]. Nevertheless, therapeutic options for either the prevention or treatment of vascular calcification are still limited, and new pharmacological targets are needed.

Soluble epoxide hydrolase (sEH), an enzyme with both epoxide hydrolase and phosphatase activities, plays an important role in regulating multiple physiological and pathological functions [3]. One of the well-investigated function of sEH is its metabolizing anti-inflammatory epoxyeicosatrienoic acids (EETs) into the inactive dihydroxyeicosatrienoic acids (DHETs) through its epoxide hydrolase activity. Using epoxide hydrolase inhibitors or deletion of Ephx2, the encoding gene of sEH, causes the increased level of EETs and shows cardioprotective properties [4]. Previously, we discover that increased sEH activity is significantly associated with severe abdominal aortic calcification [5]. But clinical studies on different polymorphisms of Ephx2 show inconsistent association with vascular calcification. The Atherosclerosis Risk in Communities (ARIC) study reports that the K55R variant of Ephx2, which results in elevated sEH activity, increases the risk of congenital heart disease [6]. Contrarily, in the Coronary Artery Risk Development in Young Adults (CARDIA) study, the R287Q polymorphism, leading to increased sEH activity, is significantly associated with great risk of coronary artery calcification (CAC) [7]. Thus, whether sEH could be a possible target for preventing vascular calcification remains uncertain.

Herein, we investigated the characteristics and potential mechanisms of sEH in vascular calcification through in vivo and in vitro experiments. We found that deletion of sEH may inhibit vascular calcification. Further gene expression analysis identified the possible role of Sirt3 in the sEH-regulated vascular calcification. Deletion of sEH may preserve the expression of Sirt3 independent of EETs hydrolysis, and thus maintain the mitochondrial adenosine triphosphate (ATP) production and morphology, significantly suppressing the vascular smooth muscle cell (VSMC) calcification. Therefore, sEH may represent a promising preventive target for CKD-related vascular calcification.

## Results

### sEH deletion attenuated vascular calcification in CKD mice

**Fig. 1:**
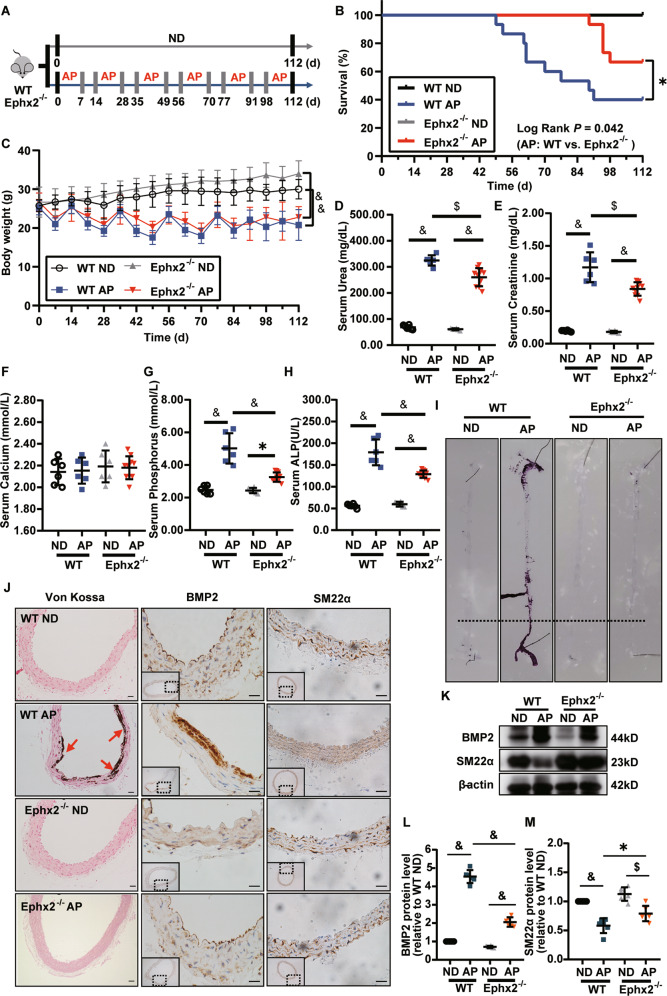
Deletion of soluble epoxide hydrolase (sEH) inhibited high adenine and phosphate (AP)-induced mouse vascular calcification. Ephx2^−^^/−^ and wild-type (WT) mice were fed with AP (*N* = 15 per AP group) or normal diet (ND) (*N* = 6 per ND group) for 16 weeks. At the end of the experiment, the numbers of living mice were 10 in the Ephx2^−^^/−^ AP and 6 in WT AP group while no death occurred in two ND groups. **A** Scheme of AP-induced chronic kidney disease (CKD) vascular calcification model. **B** Survival curves. **C** Body weights. **D** Serum urea. **E** Serum creatinine. **F** Serum calcium. **G** Serum phosphorus. **H** Serum alkaline phosphatase (ALP). **I** Alizarin Red S staining of whole mouse aortas. **J** Von Kossa staining and immunohistochemistry of bone morphogenetic protein-2 (BMP2) and smooth muscle 22 alpha (SM22α). **K**–**M** Representative western blot bands (**K**) and quantitative analysis of BMP2 (**L**) and SM22α (**M**) in VSMC. Data are presented as mean ± SD vs. WT ND: *P < 0.05; ^#^*P* < 0.01; ^$^*P* < 0.001; ^&^*P* < 0.0001. **B**, **C**: Ephx2^−^^/−^ AP: *N* = 15; WT AP: *N* = 15; Ephx2^−^^/−^ ND: *N* = 6; WT ND: *N* = 6. **D**–**H**: Ephx2^−^^/−^ AP: *N* = 10; WT AP: *N* = 6; Ephx2^−^^/−^ ND: *N* = 6; WT ND: *N* = 6.

We tested whether sEH deletion in mice attenuated vascular calcification in high adenine and phosphate (AP) induced CKD. Ephx2^−^^/−^ and wild-type (WT) mice were fed with AP diet for overall 16 weeks (Fig. 1A). As shown in Fig. 1B, sEH deletion improved survival in AP-induced-CKD mice. With the deterioration of kidney function, the body weights of both Ephx2^−^^/−^ and WT mice fed with AP were similarly decreased (Fig. 1C). The serum levels of urea and creatinine were significantly higher in AP groups after 16-week treatment (Fig. 1D, E). However, the level in Ephx2^−^^/−^ were slightly lower than that in the WT AP group. It indicated improved kidney improvement after sEH deletion. Furthermore, the serum levels of Pi and alkaline phosphatase (ALP) were significantly lower in Ephx2^−^^/−^ than in WT mice while the serum calcium was unchanged (Fig. 1F–H). As known, a high Pi and ALP level were associated with increased occurrence of vascular calcification, which was common in CKD and regarded as a contributor to cardiovascular mortality. Thus, we compared the vascular calcification in the aorta. As shown in Fig. 1I, aortic calcification indicated by Alizarin Red S staining and Von Kossa was severe in WT mice after 16-week AP diet. Osteogenic marker bone morphogenetic protein-2 (BMP2) in aorta tissue was also upregulated (Fig. 1J–L). sEH deletion completely inhibited the AP-induced aortic calcification and BMP2 upregulation (Fig. 1I, J). These data indicated a potential prevention of sEH deletion in vascular calcification of CKD.

### sEH deletion inhibited high Pi-induced VSMC calcification

**Fig. 2:**
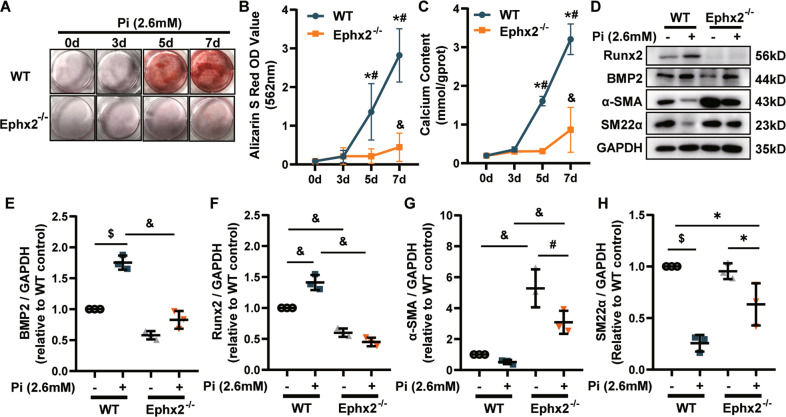
Deletion of soluble epoxide hydrolase (sEH) ameliorated high phosphate (Pi)-induced vascular smooth muscle cell (VSMC) calcium deposition and phenotypic transition. **A** Alizarin Red S staining of VSMCs. **B** The related OD value (562 nm) of Alizarin Red S staining. **C** Quantification of calcium contents of VSMCs. The calcium contents of each group were normalized to the related protein concentrations. Data are presented as mean ± SD. *P < 0.05 vs. wild-type (WT) 0d; ^#^*P* < 0.01 vs. WT 0 d; ^&^*P* < 0.0001 vs. WT 0 d. **D**–**H** Representative western blot bands (**D**), and the quantitative analysis of bone morphogenetic protein-2 (BMP2) (**E**), runt-related transcription factor 2 (Runx2) (**F**), alpha smooth muscle actin (α-SMA) (**G**), and smooth muscle 22 alpha (SM22α) (**H**) in VSMCs. Data are presented as mean ± SD. *P < 0.05; ^#^*P* < 0.01; ^$^*P* < 0.001; ^&^*P* < 0.0001.

Medial calcification is the major type of vascular calcification in CKD, among which VSMC phenotypic transition and calcium deposition are the main mechanism. Thus, we tested whether sEH deletion in VSMC could attenuate calcification. In AP-induced-CKD model, smooth muscle 22 alpha (SM22α), a contractile marker of VSMC, was markedly reduced in aortas of WT mice, whereas the level was nearly unchanged in Ephx2^−^^/−^ mice (Fig. 1J, K, M). It indicated the possible role of sEH in VSMC. Then the primary VSMCs were isolated from aortas of Ephx2^−^^/−^ and WT mice and incubated with high Pi (2.6 mM Na_2_HPO_4_) to induce in vitro calcification, separately. High Pi significantly increased the calcium deposition of WT VSMCs estimated by Alizarin Red S staining and calcium content assay in a time-dependent manner, but this increase was abolished by sEH deletion (Fig. 2A–C). Consistently with in vivo model, the expression levels of osteogenic markers, including runt-related transcription factor 2 (Runx2) and BMP2, were significantly elevated in WT VSMCs treated with high Pi compared to controls (Fig. 2D–F), whereas the expression of contractile proteins, alpha smooth muscle actin (α-SMA), and SM22α were markedly reduced (Fig. 2D, G, H). This phenotypic transition was attenuated in Ephx2^−^^/−^ VSMCs. These data supported that the deletion of sEH in VSMCs inhibited high Pi-induced VSMC calcification.

### Sirt3 was required for the inhibitory effect of sEH deletion on high Pi-induced VSMC calcification

The above data illustrated the role of sEH in AP‐induced vascular calcification, but the key molecule involved in this process was unknown. Inhibiting the epoxide hydrolase activity of sEH was an emerging therapeutic target in several diseases that shared chronic inflammation as the underlying cause. However, a recent study reported that inhibiting the epoxide hydrolase activity of sEH accelerated VSMC calcification [8]. Herein, we also used two sEH inhibitors, trans-4-(4-(3-adamantan-1-yl-ureido)-cyclohexyloxy)-benzoic acid (t-AUCB) and *N*‐[1‐(1‐oxopropyl)‐4‐piperidinyl]‐*N*′‐[4‐(trifluoromethoxy)phenyl]‐urea (TPPU), to inhibit the epoxide hydrolase activity of sEH, and to explore whether the VSMC calcification was affected. Our results showed that neither t-AUCB nor TPPU was able to decrease the calcium deposition of VSMCs estimated by Alizarin S Red staining (Supplementary Fig. 1A–D). Supplying with 11,(12)-epoxy-5Z,8Z,14Z-eicosatrienoic acid (11,12-EET) did not affect the VSMC calcification, either (Supplementary Fig. 1E, F). It indicated probably other functions of sEH but not EETs hydrolysis affecting VSMC calcification.

**Fig. 3:**
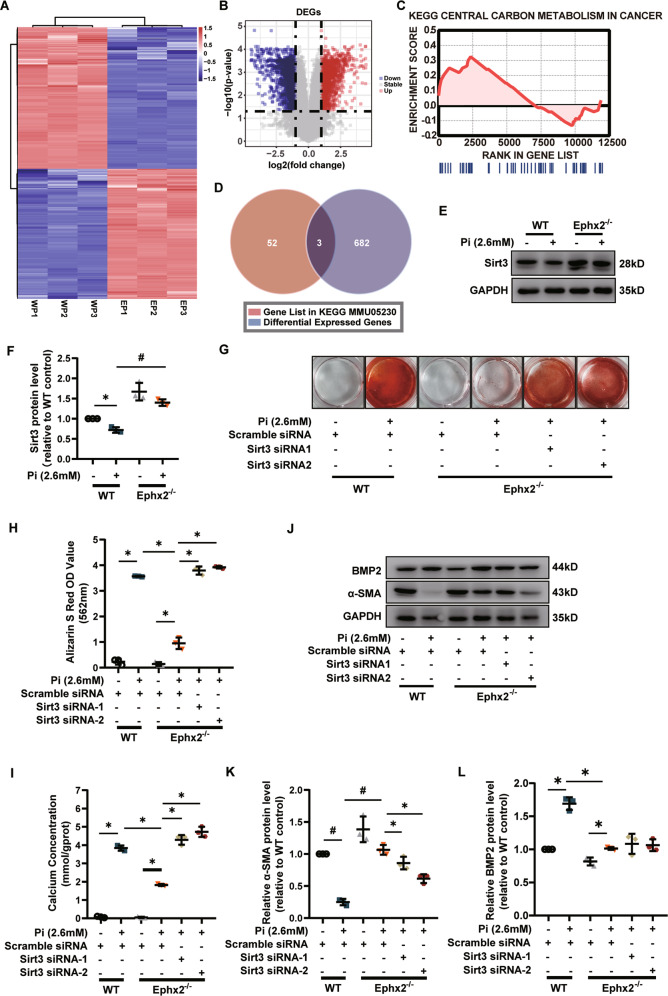
Sirtuin 3 (Sirt3) was required in the inhibitory effect of soluble epoxide hydrolase (sEH) deletion on calcium deposition in vascular smooth muscle cells (VSMCs). **A** Heatmap of all differential expression genes (DEGs) between Ephx2^−^^/−^ (EP) and wild-type (WP) VSMCs with high phosphate (Pi) treatment. **B** Volcano plots displaying the fold change (log2) of all genes analyzed. **C** Enrichment plot of Central Carbon Metabolism in Cancer gene set in gene set enrichment analysis (GSEA). **D** Venn diagram displaying DEGs in the GSEA gene sets. **E**, **F** Western blot analysis (**A**) and its quantitative analysis of Sirt3 (**B**) between Ephx2^−^^/−^ and wild-type (WT) VSMCs treated with high phosphate (Pi). **G**, **H** Alizarin S red staining (**G**) of VSMCs with scramble small interfering RNA transfection (siRNA) or Sirt3 siRNA treatment. The related staining OD values (562 nm) were shown (**H**). **I** Quantification of calcium contents of VSMCs. The calcium contents of each group were normalized to the related protein concentrations. **J**–**L** Western blot analysis and its quantitative analysis of alpha smooth muscle actin (α-SMA) (**K**) and bone morphogenetic protein-2 (BMP2) (**L**) protein expression levels in VSMCs with scramble siRNA or Sirt3 siRNA treatment, respectively, under high Pi stimulation. Data are presented as mean ± SD. *P < 0.05; ^#^*P* < 0.01; ^$^*P* < 0.001; ^&^*P* < 0.0001.

To identify the most important regulatory target genes, we performed gene expression analysis in Ephx2^−^^/−^ and WT VSMCs with high Pi treatment. Compared with WT VSMC, the gene expression analysis identified totally 796 differentially expressed genes (DEGs), among which 405 were downregulated and 391 were upregulated in Ephx2^−^^/−^ VSMCs with high Pi treatment (Fig. 3A, B). The gene set enrichment analysis (GSEA) was further performed to find out the critical KEGG pathway gene sets. As shown in Supplementary Table 1, there were eight significant signaling pathways. As known, sEH was an important enzyme for fatty-acid metabolism and related to carbon metabolism. The results of GSEA analysis included “central carbon metabolism in cancer” in the significant enrichment sets (Fig. 3C). Then the most significant DEGs in this pathway was identified by the intersection of the gene list in the GSEA and all DEGs (Fig. 3D and Supplementary Table 1). Finally, only three DEGs were left in the intersection set: Ret, Pdfga, and Sirt3. Through publication review, we found that mice with genetic deletion of sEH had increased function of Sirt3 but the specific mechanism was unknown [9]. Furthermore, Sirt3 was an important regulator for inflammatory and oxidative reaction as the intersected DEGs in other gene sets were mostly related to inflammatory and oxidative reaction. Thus, we further explored the role of Sirt3 in the sEH-related vascular calcification. The level of Sirt3 was decreased after treatment of high Pi for 7 days in cultured WT VSMCs, whereas the level of Sirt3 was unchanged in Ephx2^−^^/−^ VSMCs (Fig. 3E, F). It hinted that Sirt3 may be the potential target of sEH-mediated vascular calcification.

To further clarify the role of Sirt3 in the inhibitory effect of sEH deletion on vascular calcification, we knocked down the expression of Sirt3 via small interfering RNA transfection (siRNA) in VSMCs. Knockdown of Sirt3 abolished the inhibitory effect of sEH deletion on the calcium nodule formation and calcium deposition in VSMCs (Fig. 3G–I). Sirt3 siRNA treatment also diminished the responses of α-SMA and BMP2 expression induced by sEH deletion in high Pi-treated cells (Fig. 3J–L). All these data suggested that Sirt3 was required for sEH-mediated VSMC calcification.

### sEH deletion stabilized the expression of Sirt3

**Fig. 4:**
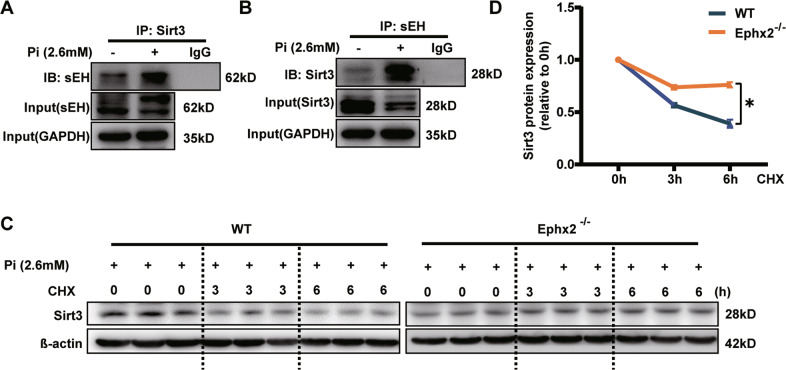
Soluble epoxide hydrolase (sEH) affected the degradation of Sirtuin 3 (Sirt3). **A**, **B** High phosphate (Pi) triggered the combination of sEH and Sirt3. Cellular lysates were immunoprecipitated (IP) with anti-sEH antibody and then immunoblotted (IB) with anti-Sirt3 (**A**), or IP with anti-Sirt3 antibody and then IB with anti-sEH (**B**). **C**, **D** High Pi treatment markedly accelerated Sirt3 degradation in wild-type (WT) compared with Ephx2^−^^/−^ VSMCs. CHX indicates cycloheximide. Data are presented as mean ± SD. *P < 0.05 vs. WT 6 h. Data are presented as mean ± SD. *P < 0.05.

Multiple studies reported that sEH was able to interact with proteins and affect their functions independent of its epoxide hydrolase activity [10, 11]. Thus, we next tested whether the protein of interaction existed between sEH and Sirt3. The result of co-immunoprecipitation (Co-IP) showed that sEH could slightly interact with Sirt3 under normal condition (Fig. 4A, B). After high Pi treatment, the sEH–Sirt3 complex was surprisingly increased (Fig. 4A, B). Previous data showed that sEH deletion was associated with increased expression of Sirt3 [9]. Thus we hypothesized that combination with sEH might lead to instability of Sirt3. Indeed, Sirt3 degraded over 50% in WT VSMCs after 6 h-cycloheximide treatment (Fig. 4C, D). But in Ephx2^−^^/−^ cells, the degradation became much slower (Fig. 4C, D). As a result, the protein level of Sirt3 was lower and further deteriorated VSMC calcification (Fig. 3G).

**Fig. 5:**
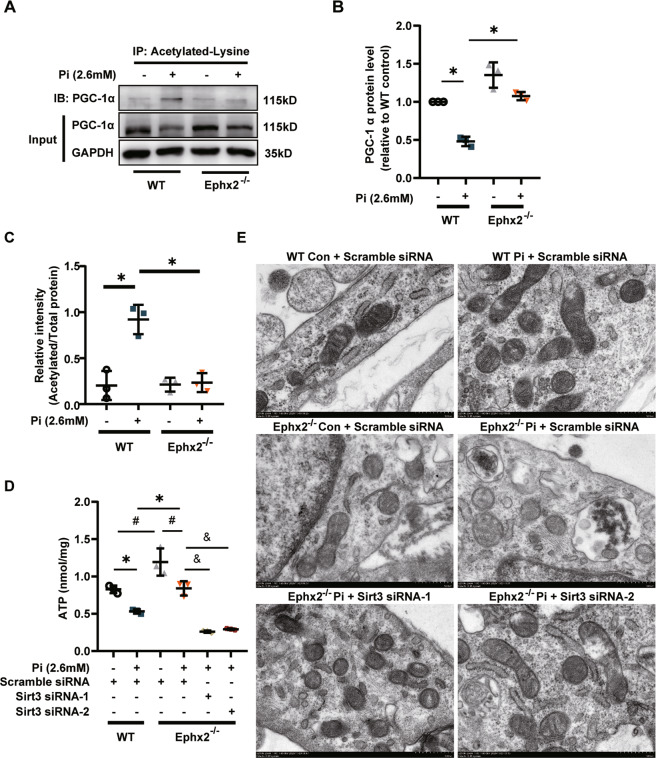
Soluble epoxide hydrolase (sEH) deletion increased Sirtuin 3 (Sirt3)-related mitochondrial adenosine triphosphate (ATP) production and improved mitochondrial morphology. **A**–**C** Western blot (**A**) and its quantitative analysis (**B**, **C**) of acetylated and total peroxisome proliferator-activated receptor-γ co-activator-1 alpha (PGC-1α) between Ephx2^−^^/−^ and wild-type (WT) VSMCs treated with high phosphate (Pi). **D** ATP detection in vascular smooth muscle cells (VSMCs) from Ephx2^−^^/−^ and WT with scramble small interfering RNA transfection (siRNA) or Sirt3 siRNA treatment, respectively, under high Pi stimulation. **E** The transmission electron microscopy (TEM) showing the fragmented mitochondrial morphology in VSMCs with scramble siRNA or Sirt3 siRNA treatment. Scale bar: 500 nm. Data are presented as mean ± SD. *P < 0.05; ^#^*P* < 0.01; ^&^*P* < 0.0001.

As known, Sirt3 exerts its function mainly in mitochondria. Peroxisome proliferator-activated receptor γ co-activator-1 alpha (PGC-1α) was the most important downstream target of Sirt3. Sirt3 was able to deacetylate PGC-1α to enhanced mitochondrial biogenesis and energy generating [12]. Herein, we observed that the protein level of PGC-1α was reduced in WT VSMCs but stable in Ephx2^−^^/−^ VSMCs after high Pi treatment (Fig. 5A, B). Furthermore, the level of acetylated PGC-1α in Ephx2^−^^/−^ VSMC was significantly lower than that of the WT VSMCs under high Pi condition (Fig. 5A, B). It indicated that the deacetylation of PGC-1α was increased in Ephx2^−^^/−^ cells. Besides, we also checked the mitochondrial ATP production between WT and Ephx2^−^^/−^ VSMCs. As shown in Fig. 5G, the ATP level was higher in Ephx2^−^^/−^ than WT VSMCs. After high Pi treatment, the ATP levels was sustained in Ephx2^−^^/−^ VSMCs while significantly reduced in WT cells (Fig. 5C). Consistently, results of transmission electron microscopy (TEM) showed that the mitochondrial morphology was severely abolished in WT VSMCs but not Ephx2^−^^/−^ VSMCs after high Pi treatment (Fig. 5D). The knockdown of Sirt3 abolished the mitochondrial improvement in Ephx2^−^^/−^ VSMCs indicating the mitochondrial changes is Sirt3-dependent (Fig. 5C, D). These data indicated that the deletion of sEH restored the expression of Sirt3, and thus ameliorated the mitochondrial dysfunction.

## Discussion

**Fig. 6:**
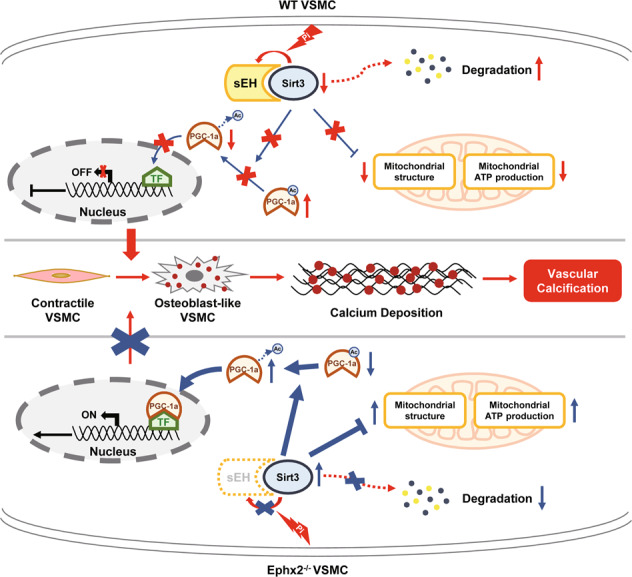
Schematic cartoon showing the mechanism of soluble epoxide hydrolase (sEH)-related vascular calcification. Under chronic kidney disease (CKD) condition, sEH interacted with Sirtuin 3 (Sirt3), leading to Sirt3 instable and degradation. This relative Sirt3 downregulation induced the increased acetylation of peroxisome proliferator-activated receptor γ co-activator-1 alpha (PGC-1α). Acetylated PGC-1α could not exert its function on gene transcription. Furthermore, the mitochondrial adenosine triphosphate (ATP) production and morphology were also impaired. All these changes triggered vascular smooth muscle cell (VSMC) phenotypic transition and calcium deposition. When sEH deletion, the sEH–Sirt3 complex could not be created and Sirt3 turned to be more stable. The sustained level of Sirt3 was able to increase the function of PGC-1α and ameliorate the mitochondrial dysfunction. As a result, VSMC phenotypic transition and calcium deposition were reduced, and vascular calcification was inhibited. TF transcription factor.

This study provided the first evidence that sEH deletion may attenuate CKD/high Pi-induced vascular calcium deposit and VSMC osteogenic transition. Furthermore, we identified Sirt3 as the possible regulator in the sEH-related vascular calcification. sEH deletion may set Sirt3 free from sEH–Sirt3 complex and stabilize Sirt3, which ameliorated mitochondrial abnormality under CKD/high Pi condition (Fig. 6).

As an important indicator of CVD, vascular calcification has been drawn great attention recently. Our team first revealed the possible link of sEH and vascular calcification by exploring the levels of sEH metabolites in patients with primary aldosteronism [5]. We discovered that increased sEH activity was significantly associated with severe abdominal aortic calcification [5]. But the possible mechanism was unknown. In present study, we found that Ephx2^−^^/−^ mice were less vulnerable to CKD-induced vascular calcification than WT ones. The VSMC from Ephx2^−^^/−^ mice also resisted to the high Pi-induced calcium deposit and phenotypic transition. All these data indicated the protective role of deletion of sEH in vascular calcification. Indeed, the protective effect of sEH inhibition on vascular remodeling has been confirmed. Previous studies demonstrated that sEH inhibition greatly reduced the increasement of cytokines, chemokines and adhesion molecules, impaired mitochondrial dysfunction, and thus suppressed the development of atherosclerosis and hypertension [13]. Furthermore, inhibition or deletion of sEH demonstrated to retain the contractile phenotype of VSMC, and inhibited VSMC proliferation and migration in the development of vascular remodeling [14, 15]. Here, we also observed that deletion of sEH restrained the decreased contractile markers SM22α and α-SMA and increased osteogenic marker BMP2 and Runx2 of VSMC. Intriguingly, a recent study reported that sEH epoxide hydrolase might play a protective role against vascular calcification [8]. Olivier Varennes et al. [8] used the sEH inhibitor t-AUCB to inhibit the epoxide hydrolase activity of sEH and was surprised to found the increased calcium deposition of rat aortic rings cultured in high Pi conditions. As known, t-AUCB was a sEH inhibitor specifically inhibiting the epoxide hydrolase activity of sEH without affecting other sEH functions like its phosphatase activity. This result simply indicated that inhibiting the epoxide hydrolase activity of sEH was likely to induce vascular calcification. However, since only using chemical inhibitor without specific knockdown of sEH, it could not fully rule out the effects of the complete sEH protein on the process of vascular calcification. In fact, several studies have reported that the effect of sEH inhibitors could be different from genetic knockout of sEH [16]. For example, despite many studies that had shown that genetic deletion of sEH decreased the pathogenicity of several diseases, both Li et al. [17] and Hutchens et al. [18] reported that deficiency of sEH in mice exacerbates cardiac dysfunction. Similarly, Benjamin Keserü et al. [19] also reported that the chronic inhibition of epoxide hydrolase activity of sEH with two chemically distinct sEH inhibitors had no obvious effect on pulmonary vascular remodeling or exercise capacity, but the genetic deletion of sEH did affect the remodeling of the pulmonary vasculature [19]. Therefore, it may be reasonable that deletion of sEH suppressed vascular calcification but not sEH inhibitors.

Beside the famous effect of EETs hydrolysis, sEH are also able to directly interact with proteins like Akt and AMP-activated protein kinase (AMPK) [10, 20, 21]. In this study, we revealed that sEH may be able to associate with Sirt3 under high Pi condition. As an important mitochondrial deacetylase, the most important biological function of Sirt3 was to deacetylate its substrates [12]. Thus, the interaction among Sirt3 and its substrates was the critical process for Sirt3 to exert its function [22]. To a certain extent, the increased association between sEH and Sirt3 under high Pi condition possibly impaired the interaction among Sirt3 and its substrates, leading to Sirt3 “dysfunction”. For example, Sirt3 was able to interact with AMPK to activate the downstream pathway to maintain bone metabolism [23] while sEH could also combine with AMPK to suppress its phosphorylation [10]. Thus, it may be possible that the interaction between Sirt3 and sEH abolish the activation of downstream pathway such as AMPK-related pathway. Although our result did indicate the interaction between Sirt3 and sEH, whether it was direct or indirect was uncertain. The exact site(s) of sEH interacting with Sirt3 and the underlying molecular mechanisms in the cross-talk between sEH and Sirt3 remained to be further uncovered, too. Nevertheless, we did observe the impaired Sirt3-related mitochondrial morphology and decreased ATP synthesis after high Pi treatment. As an important downstream target of Sirt3, PGC-1α was also downregulated and tended to be acetylated under high Pi condition. The deletion of sEH may disrupt the interaction of sEH and Sirt3, upregulate the level of Sirt3, and ameliorate the mitochondrial impairment under high Pi treatment. In fact, the relationship between sEH and Sirt3 was also reported recently [9]. Consistent with our results, a latest study proved that age-related decreased expression and activity of Sirt3 were restored in sEH null mice [9].

There are several limitations in this study. Genetic deletion of sEH mice are used, which lack both epoxide hydrolase and phosphatase activity of sEH. Our results are more likely to explain the effects of whole sEH protein rather than specific domain activity. Further, the epoxide hydrolase activity of sEH has been reported to protect against vascular calcification before [8] but here whole sEH deletion may inhibit vascular calcification. It seems that the complete sEH protein may be more important. However, there is no wildly accepted and purchasable inhibitors of phosphatase activity of sEH or the dual activities of sEH. Therefore, it may be a potential therapy of vascular calcification to explore the medicines that inhibit both functions of sEH. To this end, further investigations to describe the exact mechanisms of sEH in vascular calcification are warranted.

This work suggested the potential role of sEH deletion in the pathogenesis of vascular calcification. The results showed that deletion of sEH may prevent CKD-induced vascular calcification. Furthermore, sEH deletion possibly preserved Sirt3 expression, which was likely to abolish the impaired mitochondrial function after high Pi treatment. It is conceivable that strategies directed to inhibit both sEH functions may have preventive potential in CKD-related vascular calcification.

## Materials and methods

All methods have the corresponding literature reference. Additional protocol information is available from the corresponding author upon reasonable request.

### Animals

Ephx2^−^^/−^ mice originated on a B6;129×1/SvJ background (stock number 004165) were purchased from the Jackson Laboratory (Bar Harbor, ME, USA) [24]. This strain has been backcrossed to C57BL/6 for at least five generations. Wild-type C57BL/6 mice were served as control. All animals were housed in a temperature (25–28 °C)- and humidity (60%)-controlled animal room and maintained on a 12 h light/12 h dark cycle with food and water provided during the experiments. Animal procedures were approved by the Committee on Ethics of Animal Experiments, Sun Yat-sen University (Guangzhou, China) (Approval Number: SYSU-IACUC-2019-000359) and performed in accordance with the Guidelines for the Care and Use of Laboratory Animals published by the US National Institutes of Health (NIH Publication No. 85-23, revised 1996). All the methods were performed in accordance with approved guidelines, and all efforts were made to minimize the suffering and the number of animals used in this study.

### Animal model of chronic kidney disease

An adenine and phosphate (AP) diet-induced CKD mouse model was designed following a 16-week program as shown in Fig. 1A. The AP diet containing 0.2% adenine and 1.5% phosphate was used to accelerate the process of CKD and vascular calcification. The control groups were fed with standard chow (normal diet, ND) diet. Eight- to ten-week-old male Ephx2^−^^/−^ and WT mice were randomly divided into ND (*N* = 6 per group) and AP diet (*N* = 15 per group) groups. The 16-week distinct diets (ND vs. AP) were started following a 1-week chow diet for adaptation in all groups.

The thoracoabdominal arteries of mice were dissected to assay calcification. Serum levels of urea, creatinine, calcium, phosphorus, and ALP in the living mice were measured at the end of the 16-week experiment.

### Cell culture and cell calcification model

The primary mouse VSMC was isolated from thoracic aorta of Ephx2^−^^/−^ and WT mice aged 6–8 weeks as previously reported [25]. Briefly, after removing the adventitia and intima, the artery tissue was cut into 1–2 mm^2^ sections, suspended in fetal bovine serum (FBS) (GIBCO, 10099141 C), and plated in a culture flask. After plating for 4 h, the culture medium [high glucose DMEM (4.5 g/L glucose) (GIBCO, C11995500BT) supplemented with 20% FBS, 100 U/mL penicillin, and 100 mg/mL streptomycin (GIBCO, 15140122)] was supplemented to 5 mL, followed by static culture for 5 days. All cells were cultured at 37 °C, 5% CO_2_ in a saturation humidified incubator. Then primary VSMCs growing out of the tissue chips were trypsinized and transferred into a new flask for further experiments. Before performing further experiments, VSMCs were tested by immunofluorescence for α-SMA for phenotypic confirmation. The cells up to passages 4–8 were used for experiments. Calcification of VSMC was induced by incubation in calcifying medium [DMEM supplemented with 5% FBS, 100 U/mL penicillin and 100 mg/mL streptomycin, and Na_2_HPO_4_ (Sigma-Aldrich, 94046) at final concentration of 2.6 mmol/L] for 3–7 days as previously reported [26]. The culture medium was changed every 2 days. Treatments with t-AUCB (Cayman, 16568) and TPPU (Cayman, 11120) were used to inhibit the epoxide hydrolase of sEH. The 11,12-EET (Cayman, 50511) was used to increase the level of 11,12-EET.

### Small interfering RNA transfection (siRNA)-mediated knockdown of Sirt3

For knockdown of Sirt3 in VSMC, siRNA targeting Sirt3 was used, and a nontargeting scramble siRNA was used as a control. Two siRNA oligonucleotides (RIBOBIO) previously verified for the efficient knockdown of Sirt3 expression in VSMCs were used for final experiment (Supplementary Fig. 2). The sequences of siRNAs for Sirt3 mice genes were siRNA1: targeted GCAAGGTTCCTACTCCATA; siRNA2: targeted GCCTCTACAGCAACCTTCA. Before transfection, VSMCs were seeded in six-well plates. When met at 60% confluency, VSMCs were transfected with Lipofectamine RNAiMAX reagent (Invitrogen, 13778150) according to the manufacturer’s protocol.

### Immunohistochemical staining

Paraffin sections of mouse abdominal aortas were first placed in an oven at 60 °C for 2 h. After dewaxing using xylene and graded ethanol, the sections were soaked in sodium citrate solution and heated in a microwave oven for antigen retrieval. Then the sections were incubated in the 0.3% Triton X-100 (Sigma-Aldrich, X-100) to rupture the membrane for 20 min followed by blocking with 5% bovine serum albumin (BSA). Sections were incubated with primary antibodies against BMP2 (1:100; Abcam), SM22α (1:100; Abcam), or rabbit IgG alone (negative control) overnight at 4 °C. After incubation with an HRP-labeled anti-rabbit IgG secondary antibody, sections were stained with a DAB Kit (ZSGB-BIO, ZLI-9017). Nuclei were counterstained with hematoxylin before sealing with neutral resin.

### Western blot

Total protein samples were extracted from either tissues or whole-cell extracts with RIPA lysis buffer (Beyotime, P0013B) adding a protease and phosphatase inhibitor cocktail (Thermo Scientific, 78443). The concentrations of protein were determined by BCA Protein Assay kit (Beyotime, P0012). The protein was mixed with the 4× Laemmli Sample Buffer (BIO-RAD, 1610747) containing β-mercaptoethanol (MP, 194705) and boiled at 95 °C for 10 min. Total proteins were loaded in SDS polyacrylamide gels followed by electrophoresis and then blotted onto 0.2 μm PVDF (Merck Millipore, ISEQ00010) membranes. The membranes were then blocked by 5% BSA for 1 h at room temperature. The primary and secondary antibodies used are listed in Supplementary Table 2. Immunoblots were detected using an ECL system (Merck Millipore, WBKLS0500). The intensity of bands was quantified with ImageJ software and normalized to the WT control.

### Co-immunoprecipitation

Co-IP analysis was performed using Co-Immunoprecipitation Kit (Pierce, 26149) according to the manufacturer’s protocol. Briefly, after planned incubation, VSMCs grown in a 15 cm^2^ plate were harvested and lysed with IP lysis containing a protease and phosphatase inhibitor cocktail (Thermo Scientific, 78443). Then lysates were centrifuged, and the supernatants were collected for protein concentration determination and further Co-IP analysis. The protein samples were incubated with antibody-coupled resin overnight at 4 °C. After washing with wash buffer for three times, the IP products were eluted and added the 5× Lane Marker Sample Buffer containing 100 mM dl-dithiothreitol (MP, 194821). The samples were heated at 95 °C for 5 min and cooled to room temperature before further applying to the gel. The antibodies for Co-IP are shown in Supplementary Table 2 and used according to the manufacturer’s protocols.

### Gene expression analysis of VSMCs

Total RNA was extracted from Ephx2^−^^/−^ and WT VSMC after high Pi treatment by using TRIzol™ Reagent (Invitrogen, 15596018). About 1 μg of RNA was used to create the sequencing library by using the KAPA Stranded RNA-seq Library Prep Kit (Illumina). Then the quality of library was checked by an Aglient 2100 Bioanalyzer, and the final quantification of the library was performed by the qPCR method. The final sequencing library of different samples was denatured by 0.1 M NaOH to generate single-stranded DNA, and then amplified in situ on TruSeq SR Cluster Kit v3-cBot-HS (Illumina, GD-401-3001). The ends of the generated fragments were sequenced for 150 cycles using an Illumina X-ten/NovaSeq sequencer.

### Bioinformatics analysis

For gene expression analysis, the quantity of mRNA expression of each gene was presented as FPKM (Fragments Per Kilobase of gene/transcript model per Million mapped fragments). The DEGs, analyzed by R software (version 3.5), were identified when the log2FoldChange ≥2 or ≤−2, and the corrected *P* value <0.05. Next, GSEA were analyzed and visualized with GSEA software (version 4.0.3, http://www.broadinstitute.org/gsea/). Venn diagram was performed on the web‐tools Venn on Bioinformatics & Evolutionary Genomics site (http://bioinformatics.psb.ugent.be/webtools/Venn).

### Von Kossa staining and Alizarin Red S staining

To evaluate vascular calcification, Von Kossa and Alizarin Red S staining were performed in mice artery tissues. For Von Kossa staining, Von Kossa staining kit (Leagene, DS0003) from Leagene was used. Paraffin-embedded cross-sections were prepared. Each sample was sliced at 3 μm thickness. After deparaffinizing, samples were incubated with 5% silver nitrate under ultraviolet light for 2 h. Then un-reacted sliver was removed by incubation in 5% sodium thiosulfate for 5 min. Nuclei were counterstained with hematoxylin. For Alizarin Red S staining, the whole aorta already fixed with 4% paraformaldehyde was incubated with Alizarin Red S solution (Servicebio, G1038) for 5 min. A reddish color was displayed if positively stained.

To characterize and estimate the calcific nodules of treated VSMCs, Alizarin Red S staining was performed. After incubating with calcifying medium, the culture medium was discarded, and the treated VSMCs were washed with phosphate-buffered saline (PBS) for three times. Then 4% paraformaldehyde was used to fix the VSMCs for 30 min. After washing three times with PBS again, the fixed VSMCs were exposed to Alizarin Red S staining solution for about 20 min. At last, wash three times with PBS. Positively stained cells displayed a reddish color. To quantify Alizarin red S staining, 10% hexadecylpyridinium chloride monohydrate (CPC) (Sigma, C9002) was used for decolorization. The plate was shaken at a speed of 60 r.m.p. for 60 min at room temperature and the decolorizing solution was collected for the detection of the spectrophotometry at a wavelength of 562 nm.

### Quantification of VSMC calcification (calcium content detection)

After the incubation periods with calcifying culture medium, the treated VSMCs were incubated with 0.6 N hydrogen chloride at 4 °C. After 24-h incubation, the hydrogen chloride supernatant was collected to test the calcium content using the calcium deposition kit from Nanjing Jiancheng Bioengineering Institute (C004-2). The remaining cells were used to detect the protein concentration. The cellular calcium content standardized by protein concentration was calculated according to the formula on the instruction.

### ATP production

ATP production was detected by the Enhanced ATP Assay Kit (Beyotime, S0027) according to the manufacturer’s instructions. The results were normalized to the total protein concentrations.

### Transmission electron microscopy

After planned incubation, VSMCs were harvested and fixed by the TEM fixative (Servicebio, G1102) for 2–4 h at 4 °C. Transfer cells to centrifuge tube and spin to get cell pellet. After pre-embedded with 1% agarose solution, cell pellets were post-fixed with 1% OsO_4_ in 0.1 M PBS (pH 7.4) for 2 h at room temperature. Then the samples were dehydrated with graded ethanol and embedded with resin. The embedding models with resin and samples were moved into an oven at 65 °C to polymerize for over 48 h. The resin blocks were cut to 60–80 nm thin on the ultramicrotome (Leica), and the tissues were fished out onto the 150 meshes cuprum grids with formvar film. Then the sections were stained with uranyl acetate in pure ethanol and leas citrate. After air-drying sections at room temperature overnight, the ultrastructure was observed under a TEM (Hitachi, HT7700).

### Statistics analysis

All experiments were independently repeated at least three times. All values in the text and figures were expressed as mean ± standard deviation (SD). Data were evaluated by analysis of variance. Student’s *t*-test was applied for comparisons between two groups. One-way ANOVA was used to analyze differences between multiple groups and post hoc analysis (Bonferroni method) was performed to analyze the difference between the groups. *P* < 0.05 were considered statistically significant (*P < 0.05; ^#^*P* < 0.01; ^$^*P* < 0.001; ^&^*P* < 0.0001). Statistical analysis was performed with SPSS version 25.0 (SPSS, Inc., Chicago, IL) or Graphpad Prism version 8.0.

## Supplementary information

Supplementary Materials

## Data Availability

The datasets used and analyzed during the current study are available from the corresponding author on reasonable request.
