# Supplementary material for: Deletion of soluble epoxide hydrolase suppressed chronic kidney disease-related vascular calcification by restoring Sirtuin 3 expression
Source: Cell Death Dis. 2021 Oct 23;12(11):992. doi: 10.1038/s41419-021-04283-6 (PMC8542048; doi:10.1038/s41419-021-04283-6)
Supplement: Supplementary file 1 — Supplementary Materials [file 41419_2021_4283_MOESM1_ESM.docx]

**Supplementary Materials**

Supplementary Table 1. Genes of the intersection of GSEA KEGG pathway and DEGs.

| **Pathway** | **Gene number** | **Gene name** |
| --- | --- | --- |
| KEGG MMU05142 CHAGAS DISEASE (AMERICAN TRYPANOSOMIASIS) | 13 | Gnai1 Ccl5 Ppp2r2b C3 Nos2 Tgfb2 Ccl2 Fas Mapk13 Gnao1 Ace Tgfbr2 Il6 |
| KEGG MMU04620 TOLL-LIKE RECEPTOR SIGNALING PATHWAY | 11 | Irf5 Ccl5 Lbp Map3k8 Ctsk Spp1 Mapk13 Irf7 Il6 Ifnar2 Cxcl10 |
| KEGG MMU04623 CYTOSOLIC DNA-SENSING PATHWAY | 8 | Il18 Ccl5 Zbp1 Ifi202b Il33 Irf7 Il6 Cxcl10 |
| KEGG MMU05230 CENTRAL CARBON METABOLISM IN CANCER | 3 | Ret Pdgfra **Sirt3** |
| KEGG MMU05134 LEGIONELLOSIS | 5 | Il18 C3 Cxcl2 Il6 Cxcl1 |
| KEGG MMU05133 PERTUSSIS | 10 | C1ra Gnai1 C1s2 C1s1 C3 Nos2 Serping1 Calml4 Mapk13 Il6 |
| KEGG MMU04668 TNF SIGNALING PATHWAY | 17 | Ccl5 Socs3 Map3k8 Cxcl2 Ccl2 Fas Vcam1 Csf2 Ptgs2 Csf1 Mmp14 Junb Mapk13 Il6 Tnfaip3 Cxcl10 Cxcl1 |
| KEGG MMU05164 INFLUENZA A | 14 | Il18 Ccl5 Ddx39b Socs3 Ccl2 Fas Rsad2 Actg1 Mapk13 Il33 Irf7 Il6 Ifnar2 Cxcl10 |

Supplementary Table 2. Antibodies used in the study.

| **Antibody name** | **Brand** | **Product code** |
| --- | --- | --- |
| **Western blot** |  |  |
| α-SMA | Abcam | ab5694 |
| β-actin | Cell Signaling Technology | 8457 |
| BMP2 | Abcam | ab14933 |
| GAPDH | Cell Signaling Technology | 5174 |
| PGC-1α | Proteintech | 66369-1-Ig |
| Runx2 | Abcam | ab23981 |
| sEH | Santa Cruz Biotechnology | sc-166961 |
| Sirt3 | Cell Signaling Technology | 5490 |
| SM22α | Abcam | ab14106 |
| Anti-mouse IgG, HRP-linked | Cell Signaling Technology | 7076 |
| Anti-rabbit IgG, HRP-linked | Cell Signaling Technology | 7074 |
| **Co-IP** |  |  |
| sEH | Santa Cruz Biotechnology | sc-166961 |
| Sirt3 | Cell Signaling Technology | 5490 |
| Sirt3 | Proteintech | 10099-1-AP |
| PGC-1α | Proteintech | 66369-1-Ig |
| Acetylated-Lysine Antibody | Cell Signaling Technology | 9441 |
| Normal Mouse IgG | Sigma-Aldrich | 12-371 |
| Normal Rabbit IgG | Sigma-Aldrich | 12-370 |
| Mouse Anti-rabbit IgG (Conformation Specific) (L27A9) mAb (HRP Conjugate) | Cell Signaling Technology | 5127 |
| Anti-mouse IgG, HRP-linked | Cell Signaling Technology | 7076 |

**Supplementary Fig. 1**


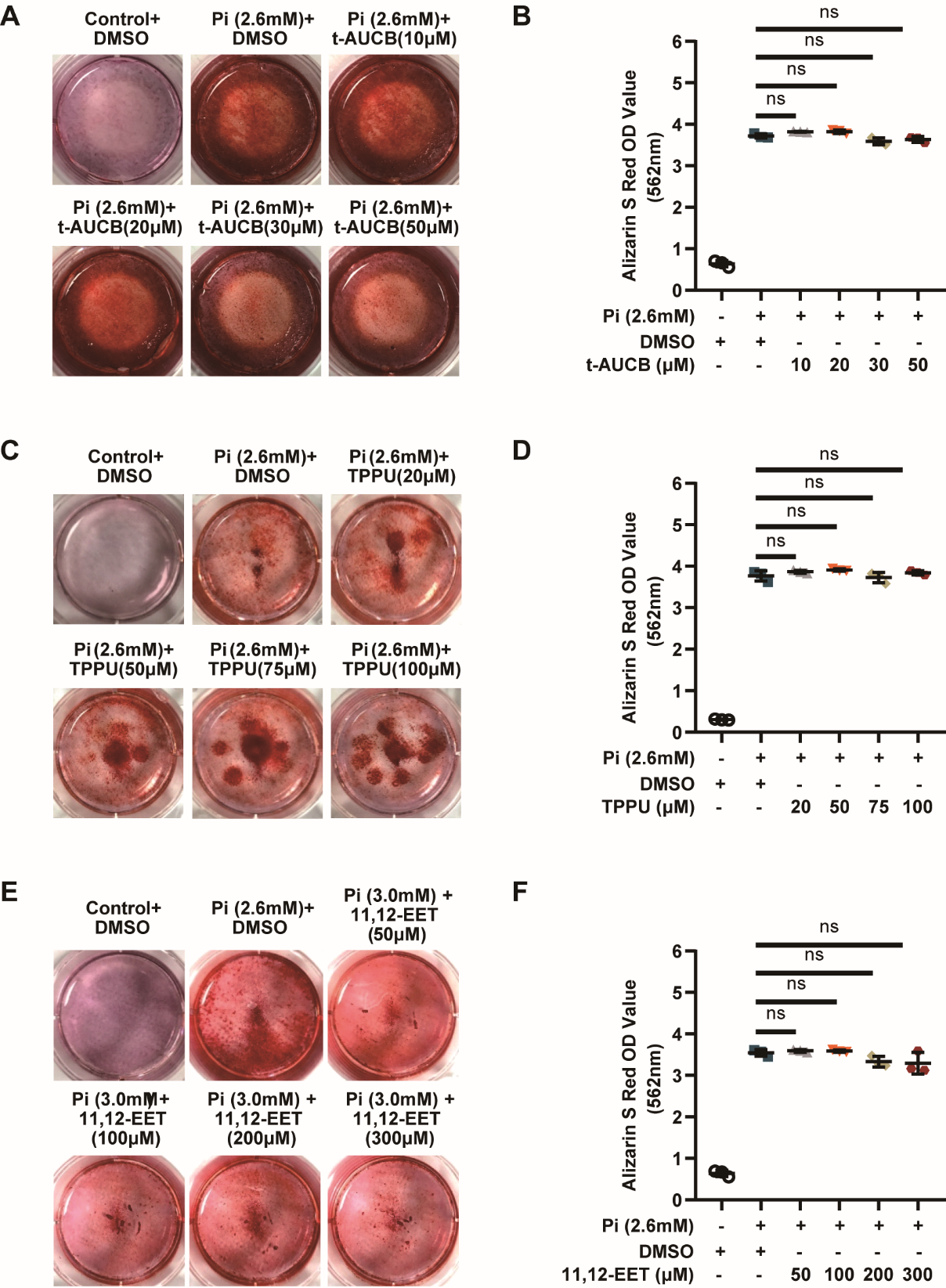


**Supplementary Fig. 1** Inhibiting the epoxide hydrolase of soluble epoxide hydrolase (sEH) did not affect vascular smooth muscle cell (VSMC) calcium deposition. Alizarin Red S staining was used to estimate the calcium deposition of VSMCs and the related OD values (562nm) were calculated. A-B. Treatment with trans-4-(4-(3-adamantan-1-yl-ureido)-cyclohexyloxy)-benzoic acid (t-AUCB). C-D. Treatment with N‐[1‐(1‐Oxopropyl)‐4‐piperidinyl]‐N'‐[4‐(trifluoromethoxy)phenyl]‐urea (TPPU). E-F. Treatment with 11,(12)-epoxy-5Z,8Z,14Z-eicosatrienoic acid (11,12-EET). Data are presented as mean ± SD. N=3. ns, non-significant.

**Supplementary Fig. 2**

**
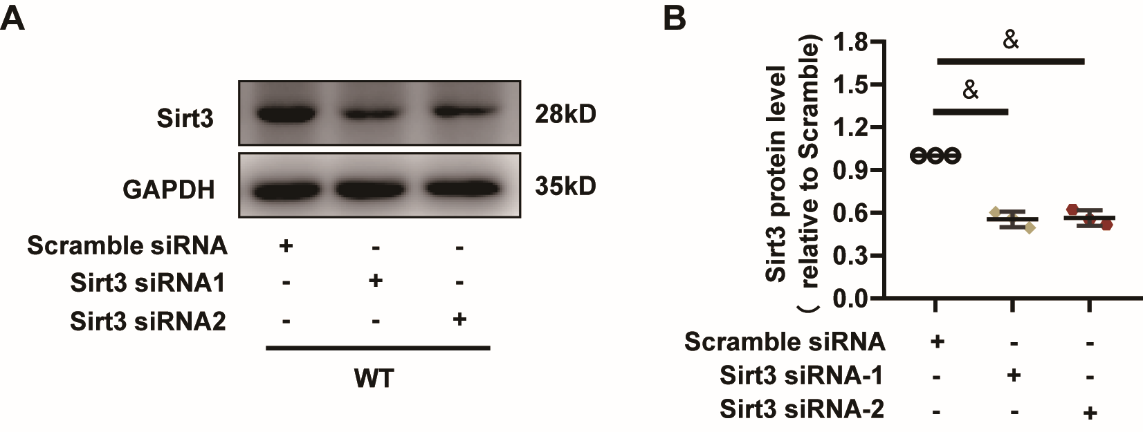
**

**Supplementary Fig. 2** The protein expression level of Sirtuin 3 (Sirt3) in vascular smooth muscle cells with scramble small interfering RNA transfection (siRNA) or Sirt3 siRNA treatment under normal condition, respectively. N=3. & *P* < 0.0001.
